# Interplay between the Formation of Colloidal Clathrate and Cubic Diamond Crystals

**DOI:** 10.1021/acs.jpcb.4c02456

**Published:** 2024-06-04

**Authors:** Łukasz Baran, Dariusz Tarasewicz, Wojciech Rżysko

**Affiliations:** Department of Theoretical Chemistry, Institute of Chemical Sciences, Faculty of Chemistry, Maria-Curie-Sklodowska University in Lublin, Pl. M Curie-Sklodowskiej 3, 20-031 Lublin, Poland

## Abstract

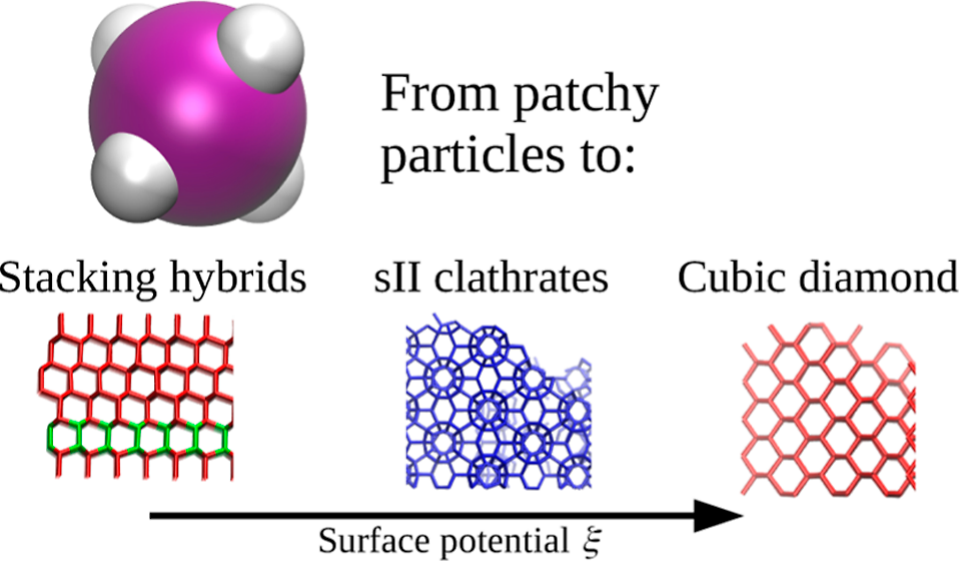

Controlling the valency of directional interactions of patchy particles is insufficient for the selective formation of target crystalline structures due to the competition between phases of similar free energy. Examples of such are stacking hybrids of interwoven hexagonal and cubic diamonds with (i) its liquid phase, (ii) arrested glasses, or (iii) clathrates, all depending on the relative patch size, despite being within the one-bond-per-patch regime. Herein, using molecular dynamics simulations, we demonstrate that although tetrahedral patchy particles with narrow patches can assemble into clathrates or stacking hybrids in the bulk, this behavior can be suppressed by the application of external surface potential. Depending on its strength, the selective growth of either cubic diamond crystals or empty sII clathrate cages can be achieved. The formation of a given ordered network depends on the structure of the first adlayer, which is commensurate with the emerging network.

## 1 Introduction

Many colloidal particle systems have been widely utilized to explain the behavior of their counterparts on the molecular scale. Studies of colloids shed light on aspects such as kinetics of crystallization^1^ and phase behavior.^2^ It was possible due to their much larger size that increases the timescales of equilibration processes, thus allowing for the measurements using an optical microscopy.^3,4^ Unfortunately, the simplest spherical colloidal particles tend to form the structures of the highest packing fraction such as face-centered cubic or hexagonal close-packed; hence, some degree of particle surface anisotropy has to be introduced in order to create open lattices such as diamond crystals. This can be realized either by changing the shape of colloidal particle or by patterning the surface with the so-called “sticky patches”. This type of particles, commonly termed patchy particles, has been extensively studied both experimentally^5−8^ and by computer simulations.^9−17^ Particular attention has been given to the particles with patches displaced in the tetrahedral arrangement as they can give valuable insight into the behavior of other tetrahedral species such as water, carbon, or silver iodide.^18−20^ However, other geometries such as triblock,^21,22^ trivalent,^23^ and octahedral,^24^ to name but a few, have also been studied.

Tetrahedral patchy particles exhibit a quite complex phase diagram^14,15^ and tend to form competing phases of interwoven hexagonal (HD) and cubic diamond (CD) polymorphs or clathrates for even more narrow patches.^10^ To date, the only successful realization of a cubic colloidal diamond crystal selectively in a single-component system required the use of tetrahedral clusters with partially embedded patches.^6^ Such particles formed only staggered bonds due to excluded volume effects that inhibited the eclipsed conformation between patches, required for the emergence of the hexagonal polymorph. Other approaches relied on the use of binary mixtures.^5,7,25^

The formation of colloidal clathrates is a formidable task. To date, it has been demonstrated with computer simulations that triblock patchy particles with triangular patches are required for the emergence of empty sII clathrate cages,^13^ which are isostructural to ice XVI.^26^ Alternatively, narrowing the spherical patches, arranged in tetrahedral symmetry, results in the formation of the same clathrate networks.^10^ Despite theoretical predictions, the experimental realization from patchy particles is yet to be achieved. Recently, He et al.^8^ argued that the particles they synthesized had too large patches, which resulted in the formation of random aggregates rather than the clathrate cages. To date, the only successful experimental realization required using hard polyhedra,^27,28^ which is a completely different class of particles.

A possible way to force the formation of crystals of the desired structure is epitaxial assembly due to the use of external fields or templates.^29−33^ For instance, Trau et al.^29^ developed an electrohydrodynamic method allowing the self-organization of multilayer colloidal crystals on the electrode surface. Such epitaxial growth can also be achieved by using patterned surfaces. However, the main problem with the latter is the necessity of using the templates to be commensurate with the assumed structure of colloidal crystals.

Therefore, in this article, we continue the investigation of tetrahedral patchy particles, for which we have recently^34^ shown that the presence of sufficiently strong external confining potential leads to the selective formation of a CD polymorph even in one-component system. We show that, similar to previous studies,^10^ controlling the valency of directional interactions is insufficient for the selective formation of desired crystalline structures in the bulk and the emergence of empty sII clathrate cages, and interwoven stacking diamonds are observed for narrow patches in a one-bond-per-patch regime. However, by applying a sufficiently strong confining surface potential, selectivity can be achieved in the formation of empty sII clathrate cages or CD crystals. This is possible because adsorbing particles forming the first layer act as a template for the developing network atop it.

## 2 Methods

### 2.1 Model Details

**Figure 1:**
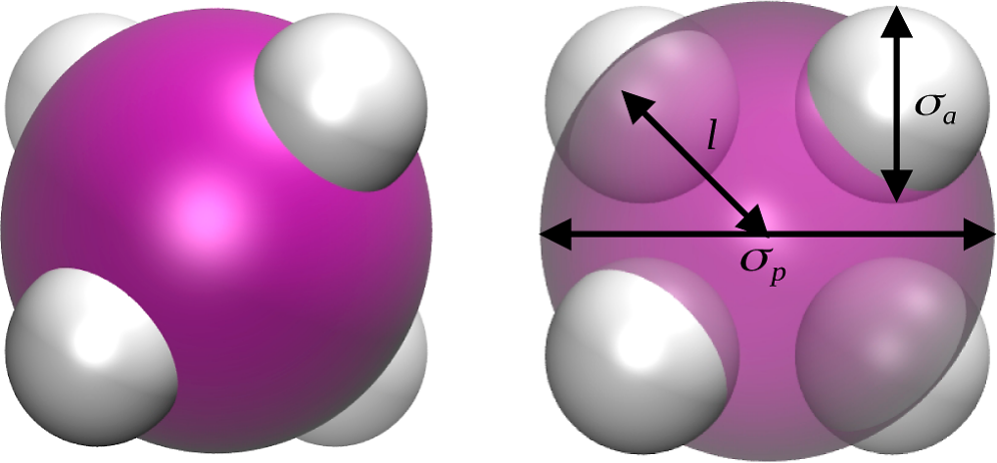
Model of tetrahedral patchy particles. The central spherical core and active sites are schematically represented (scale is not preserved) by purple and white spheres, respectively.

Tetrahedral patchy particles comprised a spherical core with diameter σ_p_ and four attractive patches of size σ_a_ embedded into its surface. The latter were manipulated by the parameter *l* (Figure 1). The patchy particles interacted via the truncated and shifted Lennard-Jones (12,6) potential, in which there is no discontinuity in both the potential and the forces.^35^ The only attraction in the system was between the active sites of patchy particles, whereas the remaining were modeled as soft-repulsive. Particle geometry has been maintained by means of the harmonic spring potential for bonds and bond angles.

On top of the interparticle interactions, we introduced an external field potential of the form of the Lennard-Jones (9,3) potential that acted solely on the core of the patchy particle
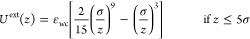
1where ε_wc_ indicates the depth of the potential well. Table 1 gives a full overview of the system parameters.

**Table 1:** Parameters of the Model^a^

parameter	symbol	value
core diameter	σ_p_	1.0σ
active site diameter	σ_a_	0.2σ
embedding distance	*l*	0.34σ
association energy	ε_aa_	5.0ε
soft-repulsive energies	ε_*ij*_	1.0ε
association cutoff	*r*_cut,aa_	2.0σ_aa_
soft-repulsive cutoffs	*r*_cut,*ij*_	1.0σ_*ij*_
bond harmonic constant	*k*_b_	1000ε/σ^2^
bond angle harmonic constant	*k*_θ_	1000ε/rad^2^
external field energy	ε_wc_	0.5ε–4ε

a In the Below *ij* = ap, pp.

### 2.2 Crystal Identification

Determination of three-dimensional crystalline networks was performed using the CHILL + order parameter, allowing for the distinction between diamond polymorphs and clathrate networks from disordered fluid. It is based on the correlation function *c*_*l*_(*i*, *j*) defined as
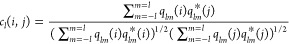
2where
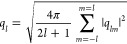
3with
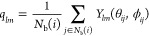
4where *Y*_*lm*_ are spherical harmonics, and for a given sphere *i*, we choose a set of its nearest neighbors, *N*_b_(*i*). Two spherical particles are connected by a bond if they are neighbors, that is, if *j* ∈ *N*_b_(*i*). For a particle *i*, the set of unit vectors **n**_*ij*_ points from *i* to particle *j* ∈ *N*_b_(*i*) in the neighborhood of *i*. Each vector **n**_*ij*_ is characterized by its angles in spherical coordinates, θ_*ij*_ and ϕ_*ij*_, on the unit sphere, evaluated between the bond and an arbitrary but fixed reference frame.

Particles in a crystalline environment were labeled by using *c*_3_(*i*, *j*). Two particles were connected by a staggered bond when *c*_3_(*i*, *j*) ≤ – 0.8 and by an eclipsed bond when −0.05 ≥ *c*_3_(*i*, *j*) ≥ – 0.2. Afterward, molecules with exactly four neighbors were further discriminated as CD (clathrate) if they were connected with one another by four staggered (eclipsed) bonds or HD, which has three staggered bonds and one eclipsed bond. Further discrimination between clathrate types has been done using *c*_6_(*i*, *j*). For more details, please see Figure S3 in the Supporting Information and the text related to it.

### 2.3 Block Analysis

To evaluate the phase diagram in the ρ – *T* plane, we have used the finite size scaling of the block density distribution functions following the method proposed by Binder.^36^ The simulation cell at distinct densities, ranging from ρ = 0.4 to ρ = 1.2, was divided into blocks of different sizes. Then, the density probability distributions for patchy particles *P*(ρ) have been estimated for each block and normalized so that the integral equals unity. The densities of coexisting phases have been taken from the maxima of these distribution functions at every considered temperature.

### 2.4 Simulation Scheme

Molecular dynamics simulations were performed in the canonical *NVT* ensemble using the LAMMPS simulation package.^37^ Trajectories were evolved with a time step of τ = 0.001 using the velocity Verlet algorithm. Temperature was maintained by the use of a Nosé–Hoover chain thermostat with a damping factor τ_NH_ = 10τ and a number of chains equal to three. The system sizes were equal to 18.8 × 18.8 × 40 or 47.8 × 47.8 × 40 for *N*_tot_ = 2500 and *N*_tot_ = 16,129 patchy particles, respectively, to check for possible finite-size effects. Such a setup corresponds to surface density *N*_tot_/(*L*_*x*_ × *L*_*y*_) ≈ 7, and the total system’s density was equal to ρ = 0.176. On the other hand, systems in the bulk were cubic boxes comprising 25^3^ = 15,625 patchy particles, the size of which depended on density ρ, which varied between 0.4 and 1.2. Such a system size was crucial for performing the block analysis, which requires as large as possible linear lengths so that one can divide the system into smaller blocks to obtain reliable results. Each of the systems was gradually cooled down from disordered states with a temperature step equal to Δ*T* = 0.01. After each run, the appearance of the nucleation event was verified using the *c*_3_(*i*, *j*) order parameter, after which the decrement in temperature was decreased to Δ*T* = 0.005. Simulations were launched for 2 × 10^8^ – 10^9^ time steps in every thermodynamic state for the equilibration. Further production runs were launched for at least 2 × 10^7^ time steps in order to collect the averages. Ten independent replicas were used for every condition to verify replica-dependent behavior.

## 3 Results and Discussion

### 3.1 Bulk Phase Results

**Figure 2:**
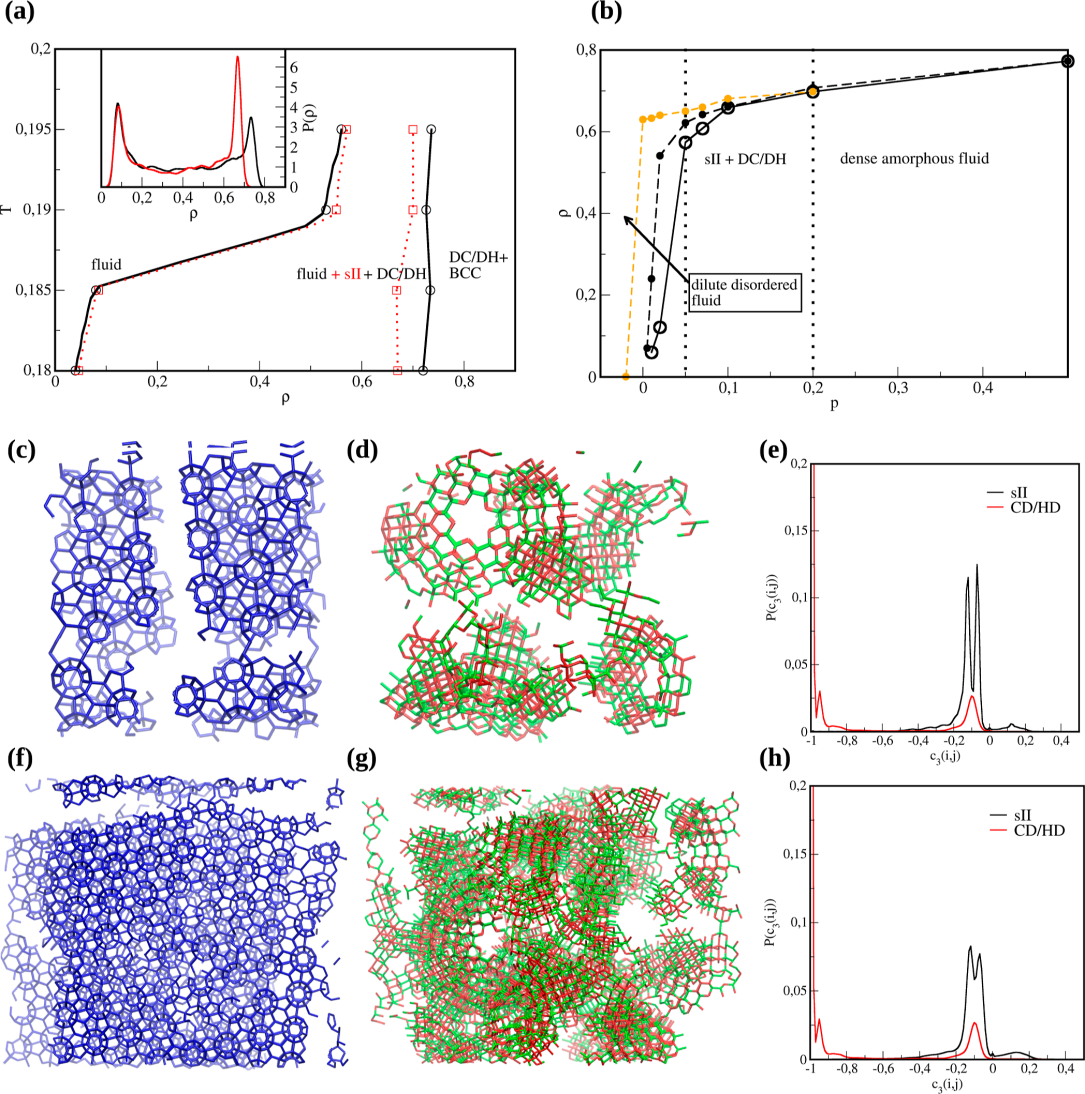
(a) Schematic bulk phase diagram for CD/HD hybrids (black) and metastable sII clathrate (red). Inset to this figure shows the corresponding block density distribution functions at *T* = 0.185 and ρ = 0.4 for these phases, evaluated for the block of linear size *L*_b_ = 8.5σ. (b) Crystallization (open symbols) isotherms and sublimation (filled symbols) evaluated at *T* = 0.180. For the latter, two starting points have been chosen, i.e., *p* = 0.5 (black dashed line) and *p* = 0.2 (orange dashed line). Vertical dotted lines divide regions with different ordered phases. Snapshots for two different replicas obtained for system size composed of 2500 (c,d) and 16,129 (f,g) patchy particles in ρ = 0.4 at *T* = 0.185. Clathrate, hexagonal, and cubic diamond particles, as identified with the CHILL + parameter, are marked in blue, green, and red, respectively. (e,h) Distribution function of the *c*_3_(*i*, *j*) parameter required for the discrimination between locally ordered environment for small (e) and large (h) system sizes.

In order to assess the influence of applied external potential, we first performed the simulations of the bulk. Part (a) of Figure 2 demonstrates a fragment of the phase diagram for tetrahedral patchy particles. Similarly to the article by Noya et al.,^10^ we observe the emergence of clathrates and the stacking hybrids of interwoven CD and HD phases. However, the contributions of different ordered phases have been found to undergo considerable changes from one replica to another as well as to depend on the system size. For this reason, we have performed the block analysis (Methods) for two independent and the most representative replicas (see the inset of Figure 2a). However, it must be emphasized that such an evaluated phase diagram is an approximate estimation of the coexistence lines. There are numerous techniques such as the direct coexistence method, thermodynamic integration, etc., devoted for the rigorous evaluation of phase boundaries.^38^ One can see that the difference between the gas branches is marginal, while for the ordered phases, it is much larger. It is noteworthy that for several replicas, we have also observed the “coexistence” between the fluid, sII clathrates, and CD/HD networks. It has to be emphasized that the temperature scale for which we have observed the formation of ordered networks is almost two times smaller in comparison to wider patches with *l* = 0.36σ^34^ (cf. Figure S2 in the Supporting Information and the text related to it). Even though in both cases, the patchy particles are within a one-bond-per-patch regime, currently, the patches being more narrow result in the effectively smaller range of the potential, shifting the temperature to lower values. This is also consistent with the previous studies.^14,15^ Moreover, the topology of the phase diagram is similar, except that in the present study, the empty clathrate sII appears, and we did not detect a “metastable” liquid region. A detailed description of how we have evaluated the phase diagram and the comparison of phase diagrams obtained for *l* = 0.34σ and *l* = 0.36σ can be found in Figure S2 and in the text related to it in the Supporting Information.

To get further insight into the phase behavior of designer tetrahedral patchy particles, we have performed additional simulations in the *NpT* ensemble at the temperature *T* = 0.18. Isotherms can be found in Figure 2b. As the pressure increases to *p* = 0.05, we observe a density jump from ρ ≈ 0.12 to ρ ≈ 0.58, and the structural analysis reveals that initially, the system forms sII clathrates; however, as the simulation proceeds, some nuclei of CD/HD diamonds start to emerge. Further compression results in a continuous increase in the density, and ultimately, most of the system consists of CD/HD stacking hybrids. When the pressure is larger than *p* = 0.2, the diamond phases disappear and dense amorphous phase start to form instead. Further compression to ρ ≈ 1.2 (*p* = 5) leads to the formation of the BCC crystal, which has also been observed in the simulations performed in the *NVT* ensemble with a system density equal to ρ = 1.2. The differences that may occur between the *NpT* and *NVT* simulations are explained in the Supporting Information.

Figure 2b also presents the sublimation curves marked with dashed lines. If the starting point was chosen to be *p* = 0.5 (amorphous state—black curve), the system at *p* = 0.2 started to nucleate into sII clathrates. Upon a decrease of pressure, the formation of diamond phases was observed. On the other hand, if the starting point was *p* = 0.2 (diamond phases—orange curve), the decrease in pressure did not change the structural behavior. Moreover, the diamond phases remained stable until eventually reaching a low-density fluid state. The hysteresis loop is also significantly larger than that in the previous case. This serves as indirect proof for the metastability of sII clathrates.

To confirm the formation of the structures described above, we have characterized them using the CHILL + parameter.^39^ It has been devised to allow for the discrimination of diamond networks, clathrates, and the corresponding interfacial particles (Methods). Selected snapshots are shown in Figure 2 for small (c and d) and large (f and g) system sizes. It is worth noticing that the clathrate structures form a one well-defined cluster with a single orientation. On the other hand, the diamond stacking hybrids do not have any preferred growth direction. Since all the faces are exposed and can simultaneously grow, many defects and grain boundaries can be seen. This possibly could be remediated by special techniques devoted to escaping local minima; however, it is outside the scope of the current study.

Figure 2e,h demonstrates the probability distributions of the *c*_3_(*i*, *j*) order parameter for two replicas, differing in the detected ordered environment for each of the system sizes. The value of the CHILL + order parameter is equal to *c*_3_(*i*, *j*) = −1 and *c*_3_(*i*, *j*) = −0.1 for staggered and eclipsed bonds, respectively (Methods). Therefore, it is evident that in the systems forming sII clathrate cages, where particles form four eclipsed bonds, there is only a characteristic peak around *c*_3_(*i*, *j*) = −0.1. On the other hand, for the diamond phases, we can see two peaks around *c*_3_(*i*, *j*) = −0.1 and *c*_3_(*i*, *j*) = −1, confirming the presence of both staggered and eclipsed conformations, characteristic of stacking hybrids. Unfortunately, the CHILL + parameter does not allow for discrimination between distinct clathrate networks. However, based on the comparison of the distribution functions of the order parameter *c*_3_(*i*, *j*) and *c*_6_(*i*, *j*) between ideal sI and sII clathrate cages and simulation results, we have identified that the formed clathrate is indeed of sII type (Figure S3 in the Supporting Information and the text related to it). The ideal clathrate structures were generated using the GenIce2 package.^40^

### 3.2 Assembly under External Field Potential

**Figure 3:**
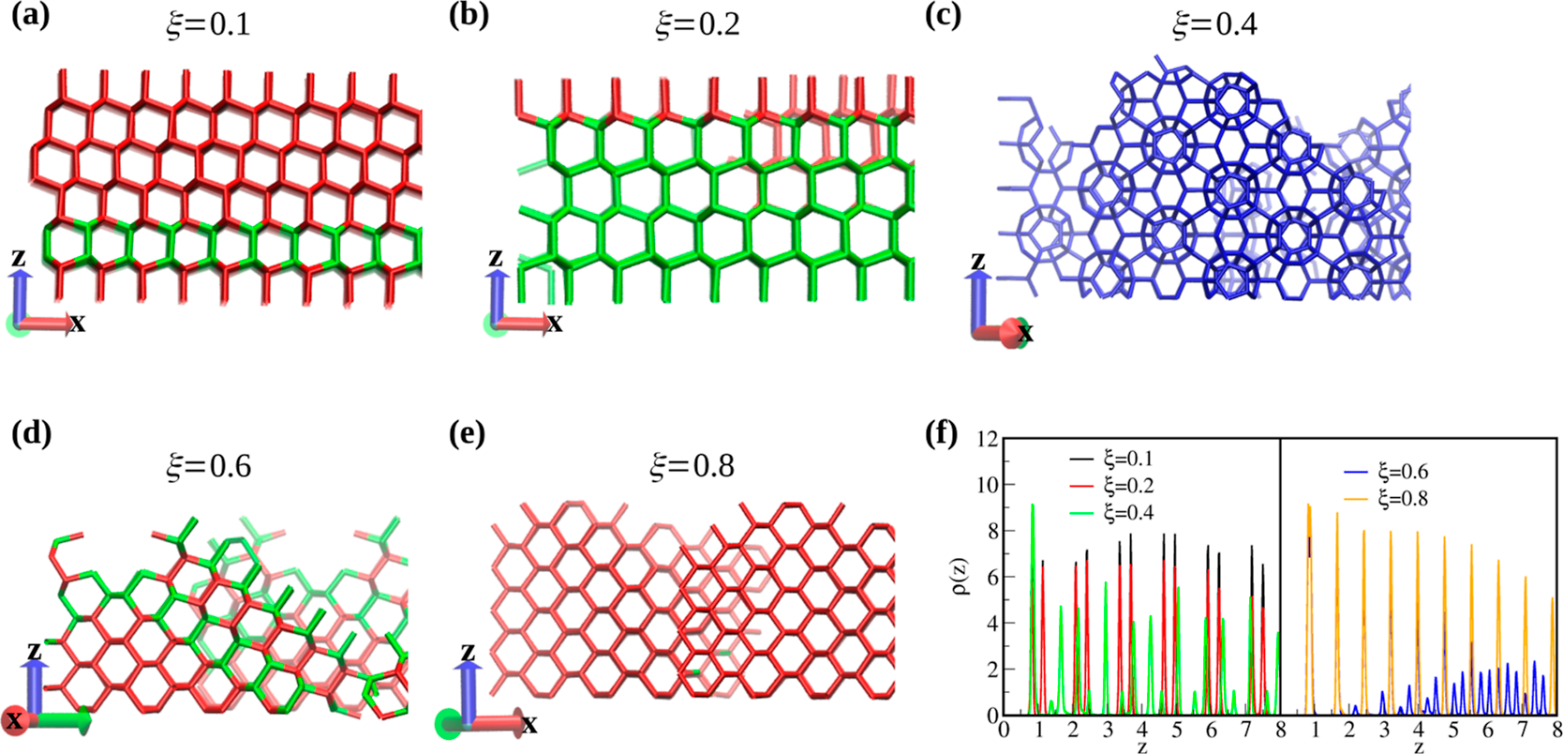
(a–e) Snapshots demonstrating different crystal growth mechanisms depending on the strength of the surface potential at *T* = 0.17 (a,b) and *T* = 0.18 (c–e). (f) Density profiles for the considered systems under different strengths of an applied external field. Red, green, and blue sticks correspond to the cubic and hexagonal diamond and clathrate environments, respectively.

Knowing the bulk behavior of currently studied tetrahedral particles with narrow patches, we are now in a position to assess the influence of the application of external surface potential on the self-assembly process. The discussion will rely on the parameter ξ = ε_wc_/ε_aa_ depicting the ratio of the strengths of particle-wall (ε_wc_) and associative particle–particle (ε_aa_) energies. The results for small system size composed of *N* = 2500 patchy particles can be found in Figure 3. For the weakest external fields, ξ = 0.1 and ξ = 0.2 (Figure 3a,b), we can see the formation of similar stacking hybrids of CD and HD networks just the same as in the bulk. For ξ = 0.1, some replicas also order into sII clathrates (Figure S4 in the Supporting Information). It is worth highlighting that the introduction of directionality facilitates the formation of a well-defined domain, with respect to the surface, which was not the case in the bulk for CD/HD phases (Figure 2-b). Surprisingly, an increase of the external field to ξ = 0.4 leads to the formation of sII clathrate networks for the majority of replicas; however, for 2 out of 10, we found similar CD/HD hybrids (Figure S4 in the Supporting Information). Further increase of the confining energy leads to similar effects, as reported in our previous article.^34^ We have observed the emergence of the CD crystals and that the stacking hybrids are not perpendicular to the *z*-axis but tilted for about 45° (ξ = 0.6, Figure 3d) or that only the CD polymorph is formed (ξ = 0.8, Figure 3e). This effect is observed for the majority of replicas; however, some exceptions have also been found for this system size (Figure S4 in the Supporting Information). As will be shown below, this can be ascribed to the frustrations that emerge from insufficiently large system size.

The density profiles ρ(*z*) shown in Figure 3f, which were calculated for systems shown in Figure 3a–e of the same figure, explain the peculiar behavior described in the previous paragraph. For very weak external potentials (ξ = 0.1 and ξ = 0.2), a characteristic bilayer structure perpendicular to the *z*-axis can be seen, indicating the (0001) or (111) faces of HD and CD, respectively. This corresponds to the stacking hybrids observed in Figure 3a,b. For ξ = 0.4, we can see that the characteristic bilayer vanishes, in favor of a single adsorption peak, and intermediate small peaks start to appear, indicating structural changes. Indeed, this corresponds to the clathrate structure, as shown in Figure 3c. Notice that the orientations of the sII clathrate structure with respect to the surface differ from one another for systems with ξ = 0.1 and ξ = 0.4 (Figure S5 in the Supporting Information). It is also worth highlighting that for two replicas for ξ = 0.4, we have observed the emergence of stacking hybrids that are tilted about 45° with respect to the surface (Figure S4 in the Supporting Information). Further increase of the external potential to ξ = 0.6 and ξ = 0.8 causes the first adsorption layer to be still well-defined; the characteristic bilayer structure vanishes and the series of peaks appear as the distance from the wall becomes larger, indicating that the crystal growth mechanism has changed again.

**Figure 4:**
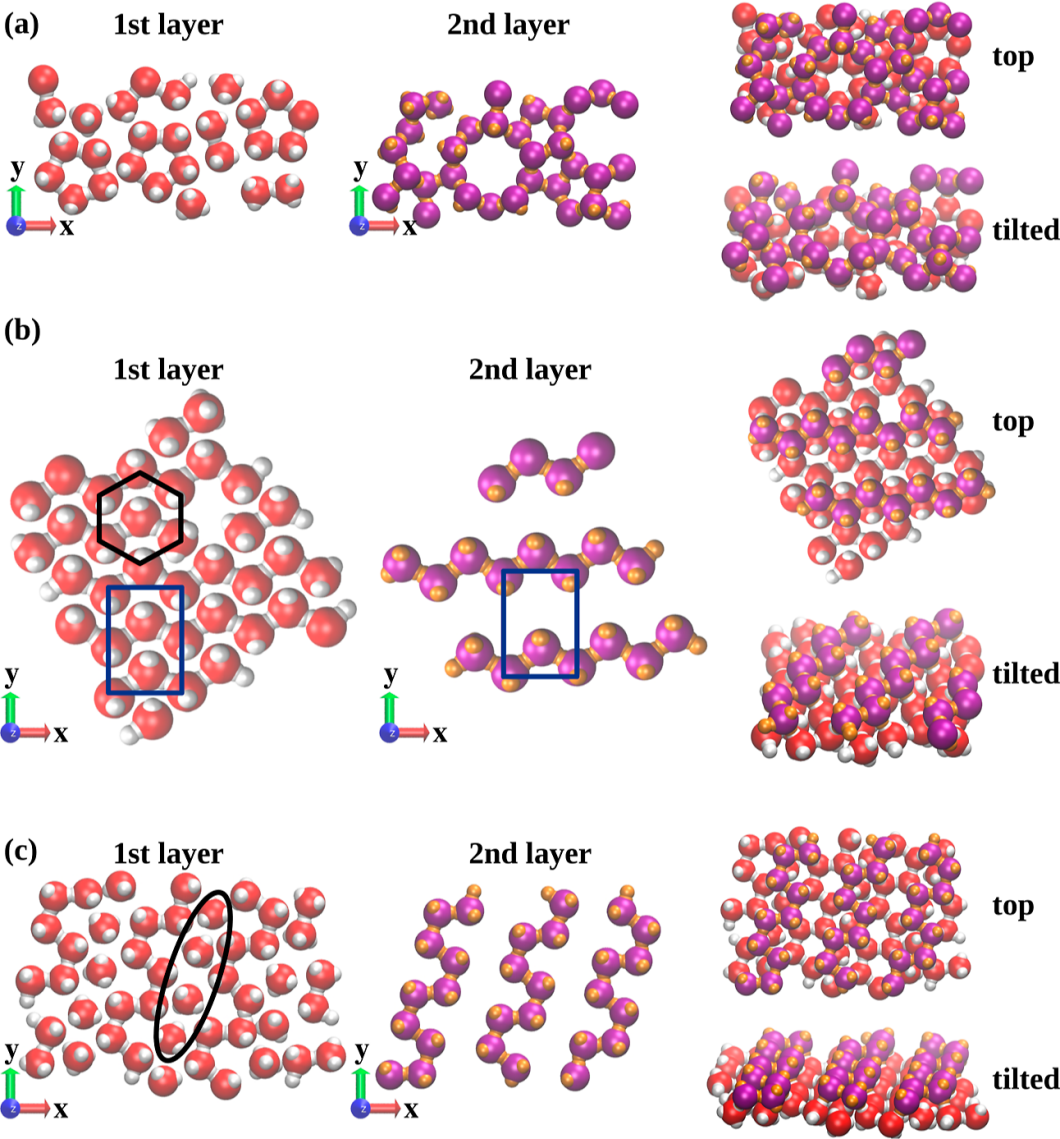
Structures of the primary adlayer (left panel) and the second adlayer (middle panel) and how they are arranged one with another from the top and tilted view (right panel) for the sII clathrates (a), cubic diamond (b), and hexagonal diamond (c).

Therefore, a naturally arising question is what is the reason for the emergence of distinct networks, depending on the strength of the external surface potential? For ξ = 0.1, we envisage that the strength of the external field is too weak to suppress the reminiscence of the bulk-like behavior, and so, both stacking hybrids and empty sII clathrates can be found. However, for the systems with larger surface potential, ξ = 0.4, it is evident from the density profiles (cf. Figure 3f) that a structural change occurs, not seen for smaller values of ξ. As already noticed, there is an emergence of a single adsorption peak, the structure of which has been visualized in the left-hand side of Figure 4a. The first adlayer formed by patchy particles tends to assemble mainly into pentagonal rings, but zigzag patterns can also be seen. The sII clathrates are composed of such pentagons as well, which can be seen in the middle panel of Figure 4a. Therefore, we conjecture that such a rather disordered structure of the first adsorbed layer promotes the emergence of empty sII clathrate cages. We have observed such behavior in all replicas where clathrates formed. The behavior that disorder promotes the formation of sII clathrates seems to support our conclusion that this structure is metastable.

Further increase of the external field results in the formation of a close-packed first layer, as visualized in the left panel of Figure 4b. We can distinguish the appearance of two unit cells—hexagonal and rectangular. The middle panel of Figure 4b shows the consecutive layers, which are the (110) faces of a growing CD. It can be readily seen that it has an identical rectangular unit cell to the triangular layer shown in the left panel. The right panel displays how these two layers are arranged with one another. This demonstrates that the close-packed triangular adlayer acts as a template and is commensurate with the (110) face of the CD polymorph. In such a way, the emergence of the stacking hybrids is avoided and the selectivity in the formation of the CD can be achieved. The same effect has already been demonstrated for tetrahedral patchy particles with wider patches.^34^

**Figure 5:**
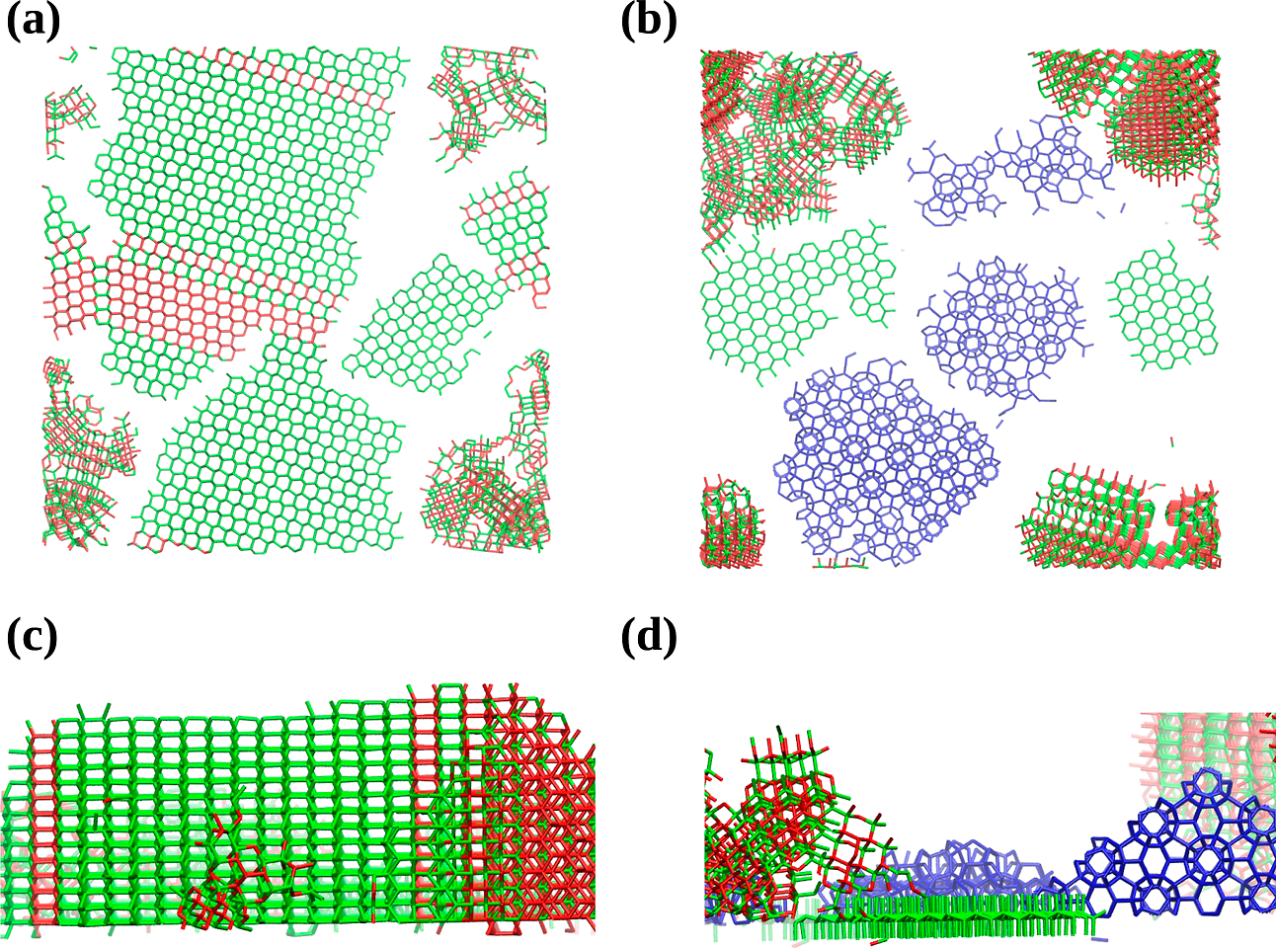
Top (a,b) and side (c,d) view of the ordered networks formed at *T* = 0.18 and ξ = 0.4 for two distinct replicas. Red, green, and blue sticks correspond to the cubic and hexagonal diamond and clathrate environments, respectively.

To further prove the metastability of clathrate networks as well as to demonstrate that the frustrations present in a smaller system result in defects of the growing networks, we have performed simulations for a larger system size, composed of *N* = 16,129 patchy particles. For this system size and with ξ = 0.4, only 2 out of 10 replicas exhibited the formation of only sII clathrate cages, indicating that emerging frustrations can be alleviated with sufficiently large system sizes. When sII clathrates have developed, the structure of the primary adsorption layer was found to be identical with that observed in a smaller system size (*N* = 2500 particles), as shown in Figure 4a. However, for the remaining replicas, the pentagonal rings have disappeared and the zigzag pattern emerged, instead. This is visualized in the left panel of Figure 4c. It is also noteworthy that some “free” particles in between the zigzag pattern have also been found (marked by an ellipse in Figure 4c). It has to be emphasized that these particles could potentially connect with the particles belonging to the zigzag pattern to form closed pentagonal rings. However, this has not been observed, suggesting that these rings are not energetically favorable and have appeared due to frustrations present in an insufficiently large system size. The consecutive layer (cf. middle panel of Figure 4c) forms the same zigzag pattern, which is a characteristic  face of the HD. Indeed, we have observed the formation of the HD polymorph instead of empty sII clathrate cages. The right-hand side panel of Figure 4 displays the spatial arrangement of the two first adsorbed layers. This again demonstrates how the structure of the first adlayer, acting as a template, can force the formation of a target network commensurate with it. Nevertheless, we have never achieved the selectivity in the formation of the HD polymorph, and the stacking hybrids always emerged, in contrary to the CD one for larger values of confining surface potential ξ. However, it has to be noticed that the contribution of the HD polymorph was always higher and equal to 62 ± 9%. An example of such a configuration can be found in Figure 5a,c.

However, we found an exception from the behavior presented above. For a single replica, a “coexistence” between all the three ordered networks—HD polymorph, CD/HD hybrids, and sII clathrate cages —was observed, as shown in Figure 5b,d. In this case, the zigzag pattern is disturbed, and the emergence of pentagonal rings is observed again, resulting in the promotion of sII clathrate cages. What is worth noticing is that it is the only case when we have observed the formation of the CD crystals right away from the surface due to the same effect as presented for larger values of ξ. Therefore, we conjecture that the formation of both HD and sII clathrate cages is a transient state for the emergence of the stable CD polymorph. The presence of imperfections and defects is caused by the fact that insufficient strength of the external surface field potential promotes the formation of the above networks.

### 3.3 Are the Obtained Networks Stable upon External Field Removal?

The ordered networks examined in the course of our study have been formed due to the presence of an external surface potential; therefore, it is instructive to evaluate how the systems behave once the field is turned off. This is particularly important as we have argued that all of the formed networks have been obtained solely because of the structure of the primary adsorption layer. However, the question remains: is it possible to remove the crystals from the surface for the postsynthetic treatment or will they collapse? To assess this, we have withdrawn the field at the lowest temperature presented here, which is *T* = 0.175, for several cases obtained for the large system size with *N* = 16,129. We have found that for the system with ξ = 0.4 that previously nucleated into the empty sII clathrate cages or the CD/HD hybrids, depending on the replica, structures remain stable even when the field is switched off. We observed the same behavior for the single crystal of CD formed in the system with ξ = 0.8. Moreover, we have found that upon the removal of the external field, ordered networks grow further. This can be ascribed to the fact that upon the removal of the external field, the particles previously belonging to the primary adsorption layer start to rearrange.

**Figure 6:**
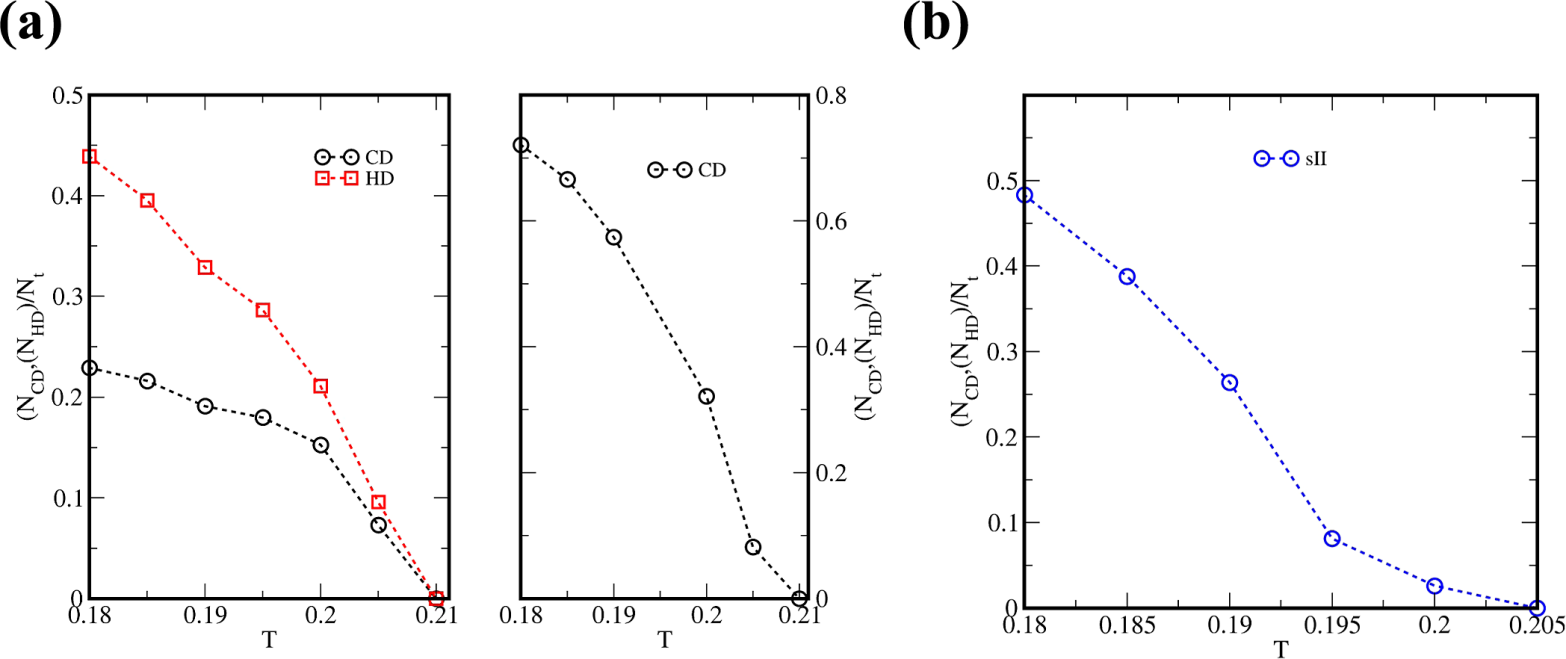
Relation of the fraction of the developed ordered networks with the temperature upon withdrawal of the external field. The initial configurations have been taken from selected replicas composed of *N* = 16,129 patchy particles that emerged for ξ = 0.4 (left panel of part a) and ξ = 0.8 (right panel of part a) and at *T* = 0.175. Part (b) demonstrates the behavior of different replica for ξ = 0.4 and at *T* = 0.175.

What is even more peculiar is that these structures do not disappear upon heating to temperatures equal to *T* = 0.205 and *T* = 0.21 for sII clathrates and diamond networks, respectively. The relation of the fraction of a given ordered structure with the temperature is shown in Figure 6. It is noteworthy that the total system density is ρ ≈ 0.176. This not only demonstrates how large the hysteresis for these systems is (cf. Figure 2a) but also indicates the possibility of “washing” these structures out from the surface for further applications.

### 3.4 Comparison of the Results for Wider Patches

So far, we have considered the tetrahedral patchy particles with narrow patches with *l* = 0.34σ. In our previous paper,^34^ we have considered wider patches with *l* = 0.36σ. Despite the particles being in the single-bond-per-patch regime, we have observed considerable differences in their behavior. Therefore, it is instructive to make a critical comparison of these two systems.

As can be seen in Table 2, the formation of empty sII clathrate cages for wider patches is not observed under any conditions, contrary to the case of *l* = 0.34σ, where they emerged in (i) the bulk and (ii) for specific strengths of externals field with ξ = 0.1 and ξ = 0.4. The comparison between the bulk phase diagrams has been already discussed in Results and Discussion and in the Supporting Information It is worth noticing that the reminiscence of the bulk-like behavior is observed up to ξ = 0.4 for both cases. Also, a selective emergence of the CD polymorph was observed over the same range of ξ in both cases. The mechanism relying on the commensurability of the primary adsorption layer with triangular symmetry is also the same, irrespective of the patch size (cf. Figure 4b). It also should be emphasized that for both patch sizes, the removal of external field has a little effect on the once-developed crystals.

**Table 2:** Comparison between the Results Obtained for Narrow (*l* = 0.34σ) and Wider (*l* = 0.36σ) Patchy Particles^a^

	the bulk	ξ = 0.1	ξ = 0.2	ξ = 0.4	ξ = 0.6	ξ = 0.8	ξ = 1.0
*l* = 0.34σ	sII or DC/DH	sII or DC/DH	DC/DH	sII or DC/DH	DC or DC/DH	DC	
*l* = 0.36σ	DC/DH			DC/DH	DC or DC/DH	DC	DC

a The latter results are taken from ref (34).

## 4 Conclusions

In this paper, we demonstrated that tetrahedral particles with narrow patches assemble into stacking hybrids of CD/HD phases or sII clathrates in the bulk and that this behavior is replica-dependent. However, this can be harnessed by the application of an external field potential. In such case, we have found that for the weak field (ξ = 0.1), structural behavior resembles that of the bulk system; however, the introduction of the directionality of the field facilitates the growth of a crystal with fewer defects than in the bulk. For the intermediate field (ξ = 0.4), an adsorption layer emerges, and its structure determines the formation of the ordered network. If the layer is triangular or forms a zigzag-like pattern, patchy particles grow into the CD and HD polymorphs, respectively. On the other hand, if the zigzag pattern is distorted, the formation of sII clathrates is promoted due to the emergence of pentagonal rings in the first adlayer. This demonstrates that the delicate interplay of the structure of the first adlayer that can be achieved by the manipulation of the surface field potential strength is a crucial dimension for the design of the desired ordered networks.

It has to be emphasized that the mechanism of the selective formation of the CD network is different from the one reported in ref (6). Here, it is the epitaxial growth that can be achieved by the use of electrode surfaces or patterned substrates.^29−33^ Noteworthily, it was only recently shown that metastable cubic ice can be obtained due to heterogeneous nucleation^41^ or due to an applied electric field^42^ for an atomistic water model. On the other hand, tetrahedral clusters with partially embedded patches used in ref (6) formed CD crystals due to the excluded volume effects. Either way, both approaches resulted in the inhibited formation of the eclipsed conformation, and selectivity is achieved in the emergence of the CD polymorph. Very recently, there has also been an experimental realization of colloidal diamond crystals from charged silica particles adsorbed on a positively charged glass substrate modified with aminopropyltriethoxysilane (APTES).^33^ The authors achieved the formation of diamond crystals due to an identical mechanism, despite using different geometries of particles. They have utilized electrostatic interactions on a flat substrate, causing the structure of such emerging first adlayer to be triangular. In consequence, it promotes the nucleation of diamond crystals in the consecutive layers.

The overall significance of this finding is that it demonstrates the possibility of selective self-assembly of sII clathrates or CD crystals from regular tetrahedral particles with spherical patches. The only requirement is the presence of a confining potential that initially increases the local density, consequently leading to the formation of ordered phases. If the strength does not prevail over the cohesive interactions, the potential will facilitate the formation of bulk-like networks, however with fewer defects than in the bulk. It has to be emphasized that the density itself would not be sufficient as the external field enforces the directionality of the growth of crystals in a given direction. On the other hand, if the external field strength is dominant, this can result in the promotion of a given structure commensurate with the first adlayer. Moreover, the problem with using prefabricated patterned surfaces being commensurate with the desired lattice structure is completely alleviated as the first adsorbed layer, formed by the tetrahedral particles, acts as a template for nucleating crystals.

## Supporting Information Available

Additional details of the model, identification and characterization of the clathrate networks, comparison of the bulk phase behavior, and additional snapshots (PDF)
