# Supplementary material for: Interplay between the Formation of Colloidal Clathrate and Cubic Diamond Crystals
Source: J Phys Chem B. 2024 Jun 4;128(23):5792–801. doi: 10.1021/acs.jpcb.4c02456 (PMC11181313; doi:10.1021/acs.jpcb.4c02456)
Supplement: Supplementary file 1 — jp4c02456_si_001.pdf [file jp4c02456_si_001.pdf]

Interplay Between the Formation of Colloidal Clathrate and Cubic Diamond Crystals

Łukasz Baran,* Dariusz Tarasewicz, and Wojciech Rżysko

Department of Theoretical Chemistry, Institute of Chemical Sciences, Faculty of Chemistry, Maria-Curie-Sklodowska University in Lublin, Pl. M Curie-Sklodowskiej 3, 20-031 Lublin, Poland

E-mail: lukasz.baran@mail.umcs.pl

Model details

In our previous work,[1] we have demonstrated that the set of parameters shown in Methods section in the main article, is efficient to describe relatively strong but reversible interactions. The parameter l (Figure 1 in the main text), depicting to what degree the active sites are embedded into the core's surface, allows for the manipulation of the patch's valency. This is because the spherical Lennard-Jones potential is screened for certain orientations of patchy particles, allowing to effectively control the extent of directionality of the interparticle interactions by the parameter l. As shown previously,[1] values larger than $l = 0.36\sigma$ allow for the creation of multiple bonds by a single active site which is an undesired effect for a current study.

In Figure S1 we demonstrate the distribution of a number of nearest neighbors (N_{nn}) around a patchy particle, calculated for the distances up to the first minimum in the radial distribution function, $r_{min} = 1.2\sigma$. The thermodynamic states were $T = 0.185$ and $T = 0.3$ for $l = 0.34\sigma$ and $l = 0.36\sigma$, respectively, for both cases in the bulk density equal to $\rho = 0.4$. It can be seen that in both cases of $l = 0.34\sigma$ and $l = 0.36\sigma$, are within the single-bond-per-patch regime. However, as reported in the previous article,[2] and demonstrated in the main document, narrowing the patches significantly changes the structural behaviour of tetrahedral patchy particles.

The bulk phase behavior

The bulk phase diagram in the $\rho - T$ plane has been evaluated using the block's analysis method proposed by Binder.[3] The difference between simulations from the NVT and NpT ensembles can be attributed to different approaches used. In the latter, the starting point for every pressure studied was a disordered and low-density configuration which after equilibration time reached the density as shown in the isotherm in Figure 1-b in the main text.

On the other hand, in the NVT ensemble two protocols have been used, depending on the system's density. For the range of $\rho = 0.4 - 0.8$ we have started from a disordered configuration at high temperature and gradually cooled down to the target temperature. This causes that some replicas crystallized into either DC/DH hybrids, sII clathrates, or their mixture. To estimate higher density branches of the phase diagram, we have selected configurations of either sII clathrates or DC/DH hybrids and compressed them to higher densities, eventually reaching $\rho = 1.2$. After compression, we have fixed the density again and performed simulations in the NVT ensemble, gathering block density distributions. We have found that DC/DH hybrids remain stable at densities higher than $\rho \approx 0.72$, and the contribution of the BCC phase increases with density. The latter was expected since the BCC network is composed of two interwoven diamond phases. On the other hand, the same protocol used for sII clathrate configuration leads to the formation of a dense amorphous fluid, as in the case of simulations in the NpT ensemble. However, it is worth noticing that in the case of the NpT ensemble, the formation of the BCC network has also been observed at the density $\rho = 1.2$.

On top of that, Figure S2 demonstrated the comparison between the phase diagrams evaluated for narrow ($l = 0.34\sigma$) and wider ($l = 0.36\sigma$) patches. It is evident that the topology of the phase diagram is similar. However, the most pronounced differences are (i) the emergence of the sII clathrates and (ii) the lack of a metastable liquid region for narrow patches. Moreover, the temperature scale is almost two times smaller for the current case.

Distinction between the clathrate networks

The CHILL+ parameter[4] allows to discriminate between different local crystalline environments including diamond and clathrate networks, based on the number of staggered or eclipsed bonds that a given particle forms. Unfortunately, using this parameter, it is not possible to differentiate between the sI and sII clathrates that are comprised of a different

number of molecules which form different types of cages. In order to avoid lengthy computations that would identify vertices and cages of clathrates,[5] we have demonstrated an alternative way based on behavior of the $c_3(i,j)$ and $c_6(i,j)$ correlation functions which enabled the distinction between sI and sII clathrate networks.

The ideal sI and sII clathrate networks were generated using GenIce2 package[6] and for each of them, we have calculated the distribution of the $c_3(i,j)$ and $c_6(i,j)$ correlation functions and compared them with the simulation results, given in Figure S3. One can see that as defined in CHILL+, the distribution functions of $c_3(i,j)$ correlation functions are almost identical for both clathrate networks and overlap with the simulation results (Figure S3 c, d). However, this is not the case for $c_6(i,j)$. The functions despite being similar for sI and sII clathrates, exhibit a systematic shift in the distributions to higher values for the sII network allowing for the discrimination. To prove that the structure formed in current simulations is sII clathrate, we have compared the distribution functions obtained from simulations with the ideal cases (Figure S3 c, d).

The formation of sII clathrates

In the main article, we have mentioned the formation of sII clathrate networks for two external potential field strengths that is $\xi = 0.1$ and $\xi = 0.4$. However, the density profiles shown in Figure S5 (a), demonstrate that these ordered clusters differ. For $\xi = 0.1$ we can see some intermediate peaks with lower intensity which are absent for $\xi = 0.4$. We have found that this is due to the different orientation of these networks with respect to the wall (cf. parts (b) and (d) of Figure S5). If one rotates the network obtained for in the system with $\xi = 0.1$ by 35° around the (0,1,0) vector, the structures appear to be identical as demonstrated in parts (c) and (d) of Figure S5). Such difference can be attributed to the formation of a primary adsorption layer for $\xi = 0.4$, which is absent for $\xi = 0.1$. Pentagonal rings which are present for the latter case promote the growth of clathrate cages in this

particular direction, commensurate with the formed structure of the primary layer.

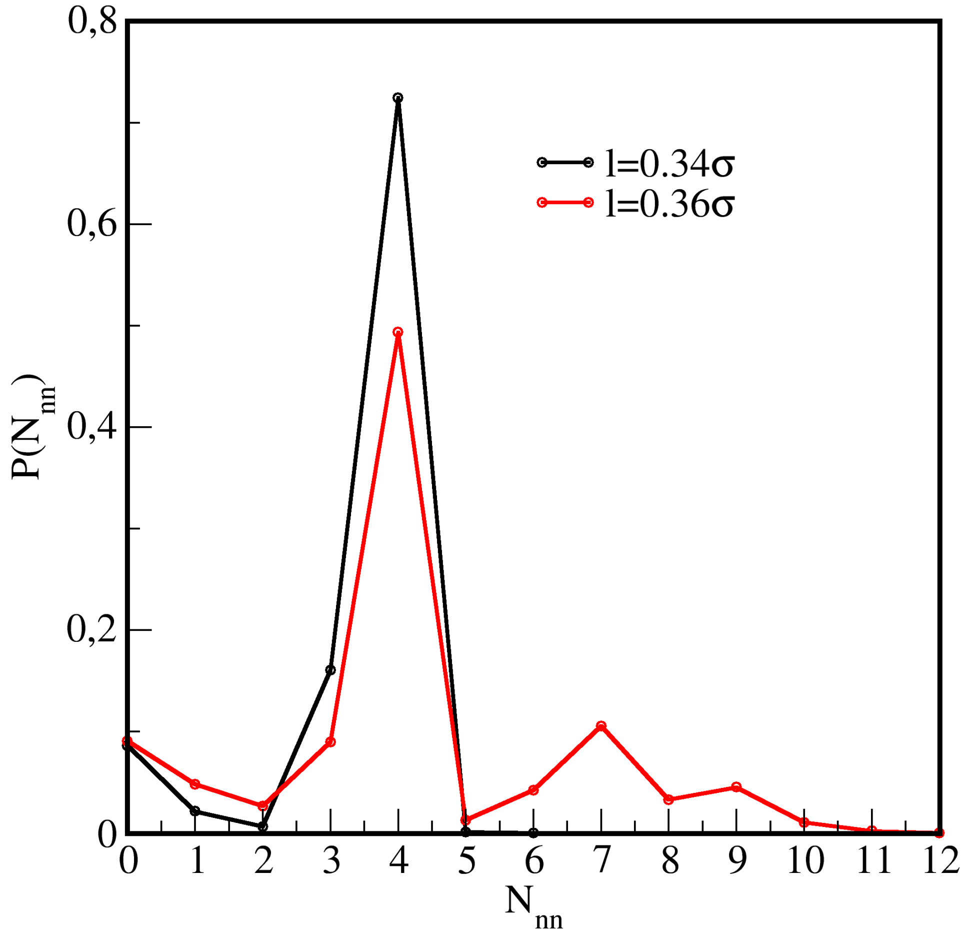

Figure S1: Distribution of the nearest neighbours (N_{nn}) for systems with embedding distance $l = 0.34\sigma$ and $l = 0.36\sigma$.

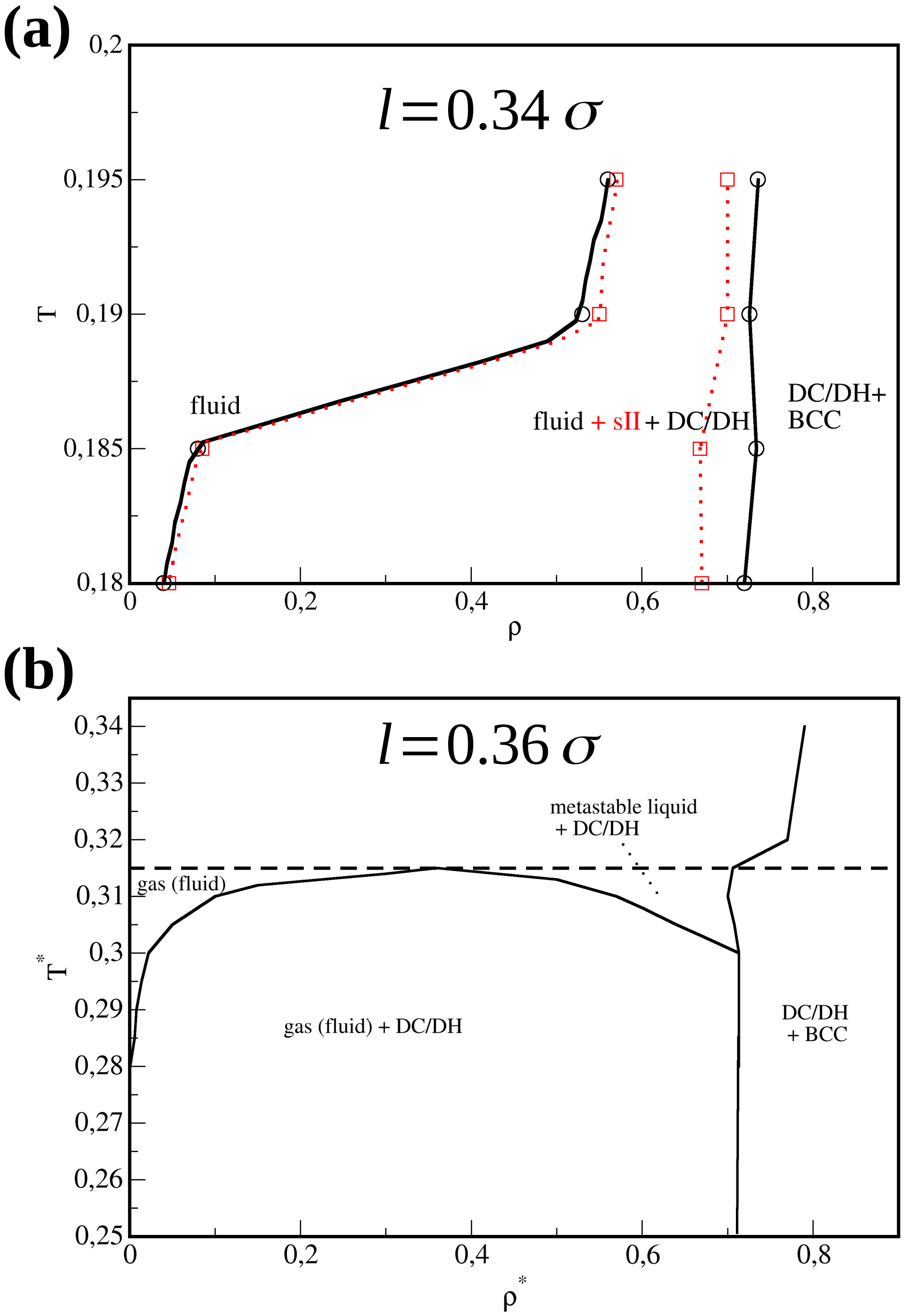

Figure S2: The comparison between the phase diagrams obtained for narrow (part a, $l = 0.34\sigma$) and wider (part b, $l = 0.36\sigma$) patches. The latter diagram is taken from Ref.[1] with permission from the Royal Society of Chemistry.

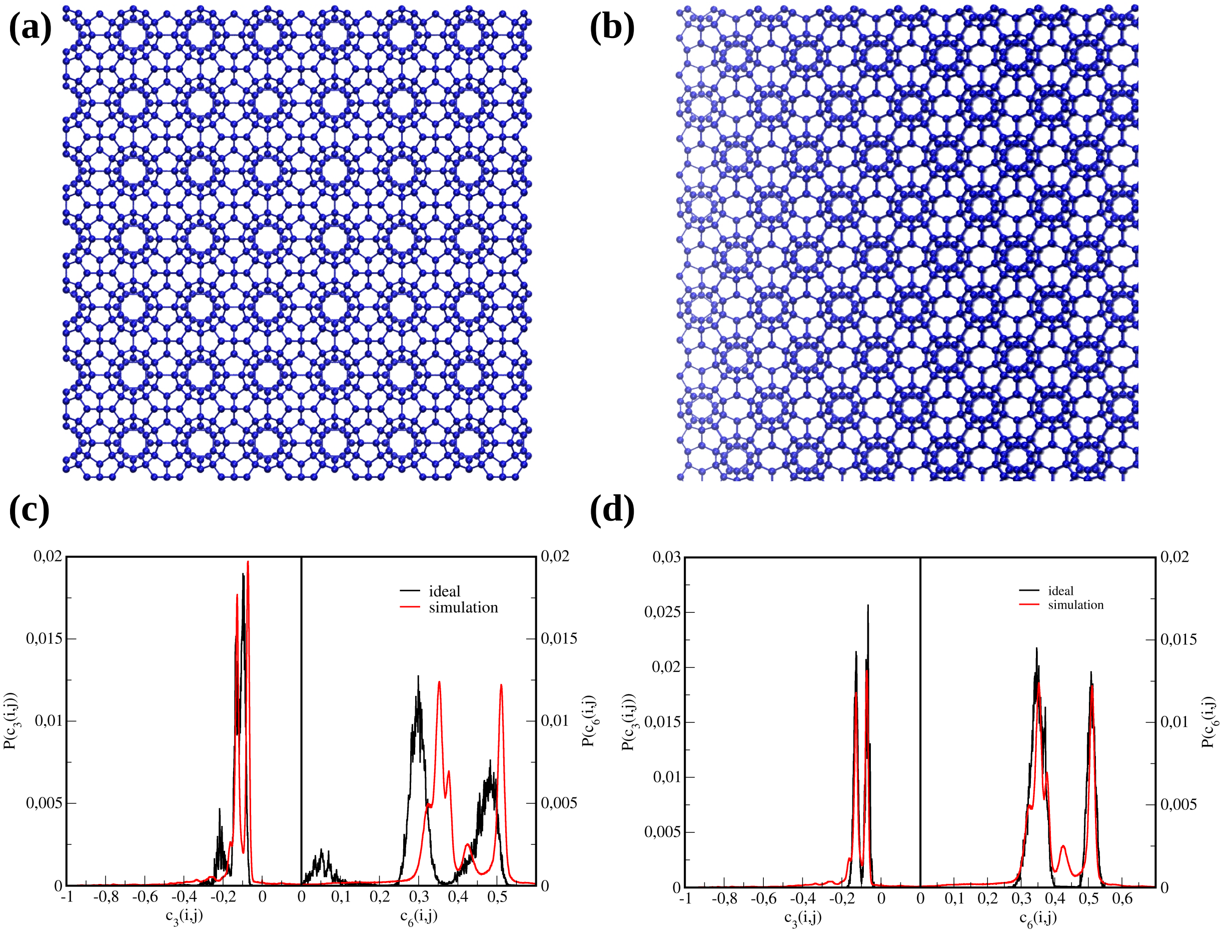

Figure S3: Parts (a, b): Ideal configurations of sI (a) and sII (b) clathrate networks, generated by GenIce2 package.[6] Parts (c, d): Distribution functions of the $c_3(i,j)$ and $c_6(i,j)$ correlation functions for sI (c) and sII (d) clathrate structures.

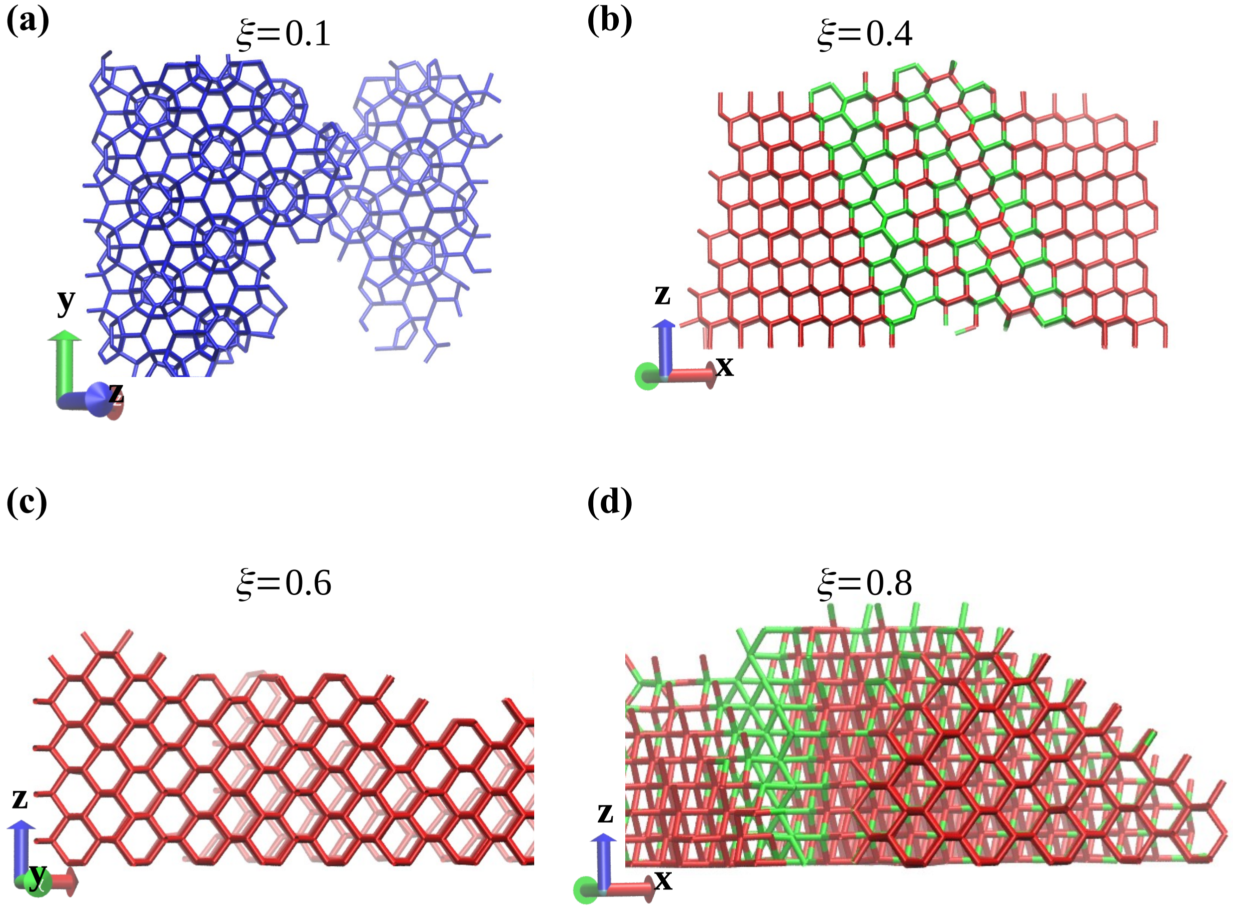

Figure S4: Snapshots of chosen replicas for different strengths of external field potential.

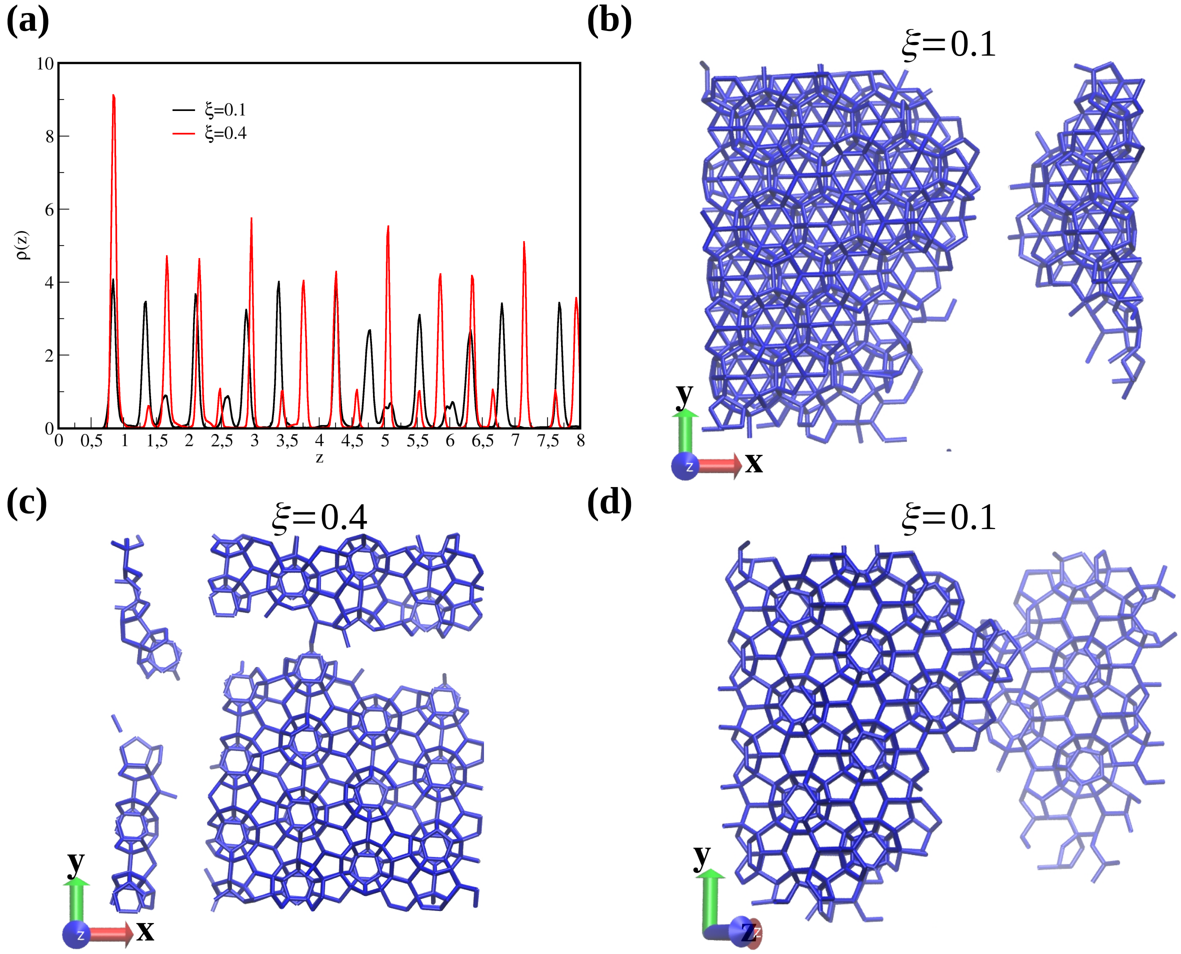

Figure S5: Part (a): Density profiles for sII clathrates for $\xi = 0.1$ and $\xi = 0.4$. Parts (b-c) Snapshots corresponding to the density profiles shown in part (a). Part (d): Configuration from part (b) tilted by 35° around the (0,1,0) vector.

References

(1) Baran, Ł.; Tarasewicz, D.; Kamiński, D. M.; Rżysko, W. Pursuing colloidal diamonds. *Nanoscale* **2023**, *15*, 10623–10633.

(2) Noya, E. G.; Zubieta, I.; Pine, D. J.; Sciortino, F. Assembly of clathrates from tetrahedral patchy colloids with narrow patches. *The Journal of Chemical Physics* **2019**, *151*, 094502.

(3) Binder, K. Finite size scaling analysis of Ising model block distribution functions. *Zeitschrift für Physik B Condensed Matter* **1981**, *43*, 119–140.

(4) Nguyen, A. H.; Molinero, V. Identification of Clathrate Hydrates, Hexagonal Ice, Cubic Ice, and Liquid Water in Simulations: the CHILL+ Algorithm. *The Journal of Physical Chemistry B* **2015**, *119*, 9369–9376, PMID: 25389702.

(5) Jacobson, L. C.; Matsumoto, M.; Molinero, V. Order parameters for the multistep crystallization of clathrate hydrates. *The Journal of Chemical Physics* **2011**, *135*, 074501.

(6) Matsumoto, M.; Yagasaki, T.; Tanaka, H. GenIce: Hydrogen-Disordered Ice Generator. *Journal of Computational Chemistry* **2017**, *39*, 61–64.
